# A Hierarchical Transcriptional Regulatory Network Required for Long-Term Thermal Stress Tolerance in an Industrial *Saccharomyces cerevisiae* Strain

**DOI:** 10.3389/fbioe.2021.826238

**Published:** 2022-01-18

**Authors:** Yuman Gan, Xianni Qi, Yuping Lin, Yufeng Guo, Yuanyuan Zhang, Qinhong Wang

**Affiliations:** ^1^ CAS Key Laboratory of Systems Microbial Biotechnology, Tianjin Institute of Industrial Biotechnology, Chinese Academy of Sciences, Tianjin, China; ^2^ University of Chinese Academy of Sciences, Beijing, China; ^3^ Institute of Marine Drugs, Guangxi University of Chinese Medicine, Nanning, China; ^4^ National Center of Technology Innovation for Synthetic Biology, Tianjin, China

**Keywords:** transcriptional regulatory network, transcription factor, RNA sequencing, long-term thermal stress tolerance, industrial strain, *Saccharomyces cerevisiae*

## Abstract

Yeast cells suffer from continuous and long-term thermal stress during high-temperature ethanol fermentation. Understanding the mechanism of yeast thermotolerance is important not only for studying microbial stress biology in basic research but also for developing thermotolerant strains for industrial application. Here, we compared the effects of 23 transcription factor (TF) deletions on high-temperature ethanol fermentation and cell survival after heat shock treatment and identified three core TFs, Sin3p, Srb2p and Mig1p, that are involved in regulating the response to long-term thermotolerance. Further analyses of comparative transcriptome profiling of the core TF deletions and transcription regulatory associations revealed a hierarchical transcriptional regulatory network centered on these three TFs. This global transcriptional regulatory network provided a better understanding of the regulatory mechanism behind long-term thermal stress tolerance as well as potential targets for transcriptome engineering to improve the performance of high-temperature ethanol fermentation by an industrial *Saccharomyces cerevisiae* strain.

## Introduction

Yeast cells are constantly exposed to various environmental stresses, either in nature or in industrial applications, such as nutrient limitation, temperature, oxidative, osmotic, chemical, and acid stresses, etc. (Boer et al., 2003; Morano et al., 2012; Takagi and Kitagaki, 2015; Song et al., 2021). To cope with these adverse situations, yeast cells have evolved remarkably sophisticated and flexible transcriptional regulatory networks that allow them to survive and thrive in harsh conditions, accompanying the induction of stress defense as well as alterations in cell growth and physiological activities (Gasch et al., 2000; Lopez-Maury et al., 2008; Ho and Gasch, 2015; Taymaz-Nikerel et al., 2016). Dissecting and understanding the transcriptional regulatory networks of yeast in response to different stresses can not only inform stress biology and disease signaling in basic research (Ho and Gasch, 2015) but also facilitate the design of strategies for improving stress resistance in strains of industrial interest in applied research (Lam et al., 2010).

From the view of application, transcriptome engineering to rationally manipulate the transcriptional states of cells provides a powerful strategy for successfully developing strains with desirable stress tolerance traits for the yeast-related industry (Lam et al., 2010; Kuroda and Ueda, 2017). Furthermore, transcription factors (TFs) are considered to be the best target for mutagenesis to achieve a desired phenotype in transcriptome engineering applications because they are not only a major component of the transcription machinery but also play a central role in transcriptional regulatory networks of the stress response (Estruch, 2000; Brent, 2016; Soontorngun, 2017). In previous studies, one general transcription factor Spt15p, a TATA-binding protein, has been broadly used for mutagenesis to enhance yeast ethanol tolerance (Alper et al., 2006; Seong et al., 2017). Recently, more transcriptome engineering applications focusing on certain stress-specific transcription factors via overexpression or site mutagenesis, such as Pdr1p and Pdr3p regulating the pleiotropic drug response and Haa1p involved in adaptation to weak acid stress, have been employed to promote tolerance against alkane biofuels (Ling et al., 2015) or acetic acid (Swinnen et al., 2017), respectively. To date, however, fewer transcriptome engineering applications have been explored for improving yeast thermotolerance, which is a suitable property for high-temperature fermentation technology (Abdel-Banat et al., 2010), partially due to a lack of a thorough understanding of master transcription factors and transcriptional regulatory networks specifically involved in the response to long-term thermal stress instead of the heat shock response under short-term thermal stress (Gao et al., 2016).

To investigate the global cellular response of *S. cerevisiae* to heat or other stresses, two basic approaches have been used. One approach is transcriptome analysis to study the genome-wide reprogramming of gene expression, which thereby helps to prioritize master transcription factors that are involved in the control of differential gene expression under stress conditions (Gasch et al., 2000; Causton et al., 2001; Ma and Liu, 2010). In this way, the heat shock transcription factor Hsf1p and the general stress transcription factors Msn2p and Msn4p (Msn2p/4p) were well identified to be primary modulators of the heat shock response to govern heat-induced transcription of heat shock protein (HSP) genes in *S. cerevisiae* (Verghese et al., 2012). Another approach screened the *S. cerevisiae* deletion strain collection to identify single gene deletions that influence cell survival at different levels of heat stress (Zakrzewska et al., 2011; Gibney et al., 2013; Jarolim et al., 2013). These studies on genome-wide libraries of yeast deletion strains revealed that the genes required for heat-shock survival have little overlap with the heat-activated or repressed genes, and a small portion of them are regulated by the heat-shock transcription factor Hsf1p (Gibney et al., 2013; Jarolim et al., 2013). The cell division transcription factors Swi6p and Hac1p, which are involved in the unfolded protein response, were found to play roles in the maintenance of heat shock resistance (Jarolim et al., 2013). In contrast to the above studies focusing on the heat shock response under short-term thermal stress, our recent studies indicated that *S. cerevisiae* has a distinct regulatory mechanism of thermotolerance under long-term thermal stress through proteomic surveys and identified 23 transcription factors whose transcriptional expression was uniquely induced in response to prolonged thermal stress compared with the heat shock response (Shui et al., 2015; Xiao et al., 2018).

In the present study, we further compared the effect of the single-gene deletion of twenty-three previously identified transcription factors on cell growth and physiological activity under prolonged thermal stress with those on heat shock survival, thus unravelling three specific transcription factors—Sin3p and Srb2p—required for maintaining physiological activities of yeast cells under long- but not short-term thermal stress. Additionally, the deletion of *MIG1*, which was previously identified to be the most significantly upregulated specific transcription factor at prolonged thermal stress (Xiao et al., 2018), showed no apparent effect on cell growth and physiological activity at prolonged thermal stress or heat shock survival. Next, aiming to uncover how these three core and specific TFs could be involved in regulating transcriptional responses to long-term thermal stress in similar or different ways, the genome-wide transcriptome profiles of single TF deletion strains of *SIN3*, *SRB2* and *MIG1* grown at high temperature were analyzed and compared by next-generation sequencing (RNA-seq) using the wild type strain as a reference. Eventually, a hierarchical transcriptional regulatory network centered on these three transcription factors, was illustrated to be required for the long-term thermal stress tolerance of *S. cerevisiae*.

## Materials and Methods

### *S. cerevisiae* Strains

The strains used in this study are listed in Supplementary Table S1. Strain ScY01a, a strain of mating-type a, was derived from an adaptively evolved thermotolerant strain ScY01 (Shui et al., 2015) and used as host strains as specified in the text. ScY01 is an industrial strain. To disrupt key transcription factors (TFs) in strain ScY01a, three approaches based on PCR amplification and one-step gene replacement were employed depending on which one could work efficiently in our strain (Guldener et al., 1996). The gene disruption cassettes containing the *Kan*MX expression module flanked by homologous sequences to the target TF were either obtained using fusion PCR or direct PCR amplification from the yeast knockout collection (BY4743 deletion collection, EUROSCARF, Frankfurt, Germany). To delete the whole ORF of *ABF1*, *FHL1, MIG1, PDR3, RAP1, RLM1, SIN3, SRB2, STB5* and *YHP1*, the *KanMX* expression cassettes were amplified using TF-specific primers from the plasmid pUG6 (Guldener et al., 1996), and fused with approximately 500-bp homologous sequences located upstream and downstream of the TF ORF that were PCR amplified from ScY01a genomic DNA (for primers, see Supplementary Table S2). To disrupt the ORF of *SK O 1*, *SKN7*, *SNF2*, *SOK2* and *YAP1*, the TF-specific *Kan*MX expression cassettes were fused with approximately 500-bp homologous sequences located at the beginning and end of the TF ORF that were PCR amplified from ScY01a genomic DNA (for primers, see Supplementary Table S2). To knock out *ASH1, CBF1, CDC73, CST6, GCR2, ISW2, MBP1, SWI4, ACE2, TEC1 and SFP1*, their null alleles, marked by *Kan*MX, were amplified from the yeast knockout collection (BY4743 deletion collection, EUROSCARF, Frankfurt, Germany) (Wang and Chen, 2015). All the gene disruption cassettes were separately transferred into the ScY01a background using the electrotransformation method (Becker and Guarente, 1991). Positive transformants were selected on G418 selective plates, which contained 10 g/L yeast extract, 20 g/L tryptone, 20 g/L of glucose, 20 g/L agar and 400 μg/ml of geneticin G418. To confirm successful deletion, diagnostic PCR reactions with primers (see Supplementary Table S2) targeting approximately 200 bp upstream of the TF gene and the KanMX-specific primer (KanMX-vf-Rv) were used. Following the previously reported method of overexpressing *SRB2* and *MIG2* (Xiao et al., 2018), *SIN3* overexpression was performed using the low-copy commercial plasmid pRS316, and was driven by its original promoter and a constitutively strong promoter of *TEF1*, respectively.

### Micro-Aerobic Fermentation

Flask fermentations were performed under micro-aerobic conditions at 40°C for 36 h and at 30°C for 24 h, where cells were grown with shaking at 220 rpm in 100 ml flasks containing 50 ml fermentation media. Samplings were conducted every 4–6 h. YP medium (10 g/L yeast extract, 20 g/L tryptone) containing 200 g/L glucose was used as the fermentation medium. To prepare seeds, yeast cells on G418 selective plates were grown in 50 ml flasks containing 20 ml YP media with 200 g/L glucose at 30°C overnight (∼15 h). Cells were harvested by centrifugation and then inoculated into fermentation media. The initial OD_600_ used for micro-aerobic fermentation was 0.5.

### Spot Tests for Heat-Shock Survival

Heat-shock assays and spot assays of cell survival were performed as described previously with some modifications (Gibney et al., 2013; Jarolim et al., 2013). Yeast cells were grown in 10 ml tubes containing 3 ml YP medium with 200 g/L glucose at 30°C with shaking at 220 rpm overnight (∼15 h). Cells were harvested by centrifugation and inoculated into 25 ml YP medium with 200 g/L glucose in 50 ml flasks, to achieve an initial OD_600_ value of 0.2. The cell cultures were grown to early log phase at 30°C with shaking at 220 rpm for 5 h. Two aliquots containing an appropriate amount of cells were harvested and resuspended in 1 ml supernatants to obtain cell suspensions with an OD_600_ of 5.0. One aliquot of cell suspension was placed on ice as a preheat shock control. The other aliquot of cell suspension was transferred to a 10 ml tube, and incubated at 50°C for 30 min with shaking at 200 rpm, and immediately chilled on ice for 5 min. Both preheat shock and heat-shock aliquots of cell suspensions with an OD_600_ of 5.0 were diluted to OD_600_ of 1.0, 0.3, 0.1, 0.03, 0.01, and 5 µl samples at each dilution spotted on YPD plates (YP medium with 20 g/L glucose), which were incubated at 30°C for 48 h.

### RNA Sequencing and Data Analysis

Three key TF deletion strains, ScY01a (*sin3∆*), ScY01a (*srb2∆*) and ScY01a (*mig1∆*), as well as the wild-type strain ScY01a (Supplementary Table S1) were subjected to transcriptome analysis using RNA sequencing. Micro-aerobic cultures of these four strains with an initial OD_600_ of 0.2 were carried out at 40°C in biological duplicates using 50 ml YP medium with 200 g/L glucose in 100 ml flasks with shaking at 220 rpm. After cell incubation for 10 h to early log phase, cells were harvested in Falcon tubes precooled in liquid nitrogen by centrifuging for 5 min. Due to the experimental setup issue, culture samples grown at 30°C were prepared in a different batch of experiments. The supernatant was removed, and the pellet was stored at −80°C until further use.

RNA extraction and sequencing libraries were prepared and sequenced on the Illumina HiSeq platform using 150-bp paired-end sequencing by Genewiz Inc. (Suzhou, Jiangsu, China). The *S. cerevisiae* S288c genome was used as a reference, and downloaded from RefSeq at NCBI (sequence assembly version R64, RefSeq assembly accession: GCF_000146,045.2) including 16 chromosomes and the mitochondrial genome (www.ncbi.nlm.nih.gov/refseq/). An average of 17.8 ± 0.4 million cleaned reads and an average mapping rate of 63.4 ± 4.0% corresponded to approximately 140-fold coverage of the reference genome (total size of 12.17 Mb) were generated for each library. The cleaned reads were aligned to the reference transcriptome by Bowtie (version 2.2.3) (Langmead et al., 2009). Reads per kilobase of exon model per million mapped reads (RPKM) of each gene were calculated based on the length of the gene and reads count mapped to this gene (Trapnell et al., 2010). Then, the transcript quantification was estimated from mappings by RSEM (Li and Dewey, 2011). The R package DEseq2 was used in the differential expression analysis (Wang et al., 2010). RNA sequencing data have been submitted to the Gene Expression Omnibus (GEO) under GEO accession nos. GSE104356 and GSE148213. Differential gene expression (DEG) in comparing the key TF deletion strains with the wild-type strain ScY01a was analyzed as previously described (Conesa et al., 2016). Significantly differentially expressed genes (SDEGs) in the comparison of the TF deletion strain versus the wild-type strain at the same temperature condition were then extracted by applying an absolute fold-change threshold of 2.0 or greater and a false discovery rate (FDR)-corrected cutoff *p*-value of 0.05 or less (Supplementary Table S3). SDEGs from different fermentation temperatures, including 40°C and 30°C, were further subjected to Venn diagram analysis (Venny 2.1.0, https://bioinfogp.cnb.csic.es/tools/venny/) (Supplementary Table S3). All the above SDEG lists were further tested for Gene Ontology (GO) biological process enrichment using FunSpec with a *p*-value cutoff of 0.05 and Bonferroni correction (Robinson et al., 2002) (Supplementary Table S4).

### Analysis of Transcription Regulatory Associations

To dissect transcription factors and transcription regulatory networks downstream and upstream of the core TFs, including Sin3p, Srb2p and Mig1p, the YEASTRACT database (http://www.yeastract.com/) (Teixeira et al., 2006; Teixeira et al., 2014) was used. The tools of Search for Genes (http://www.yeastract.com/formfindregulated.php), Search for Associations (http://www.yeastract.com/formregassociations.php), Rank by TF (http://www.yeastract.com/formrankbytf.php) and Search for TFs (http://www.yeastract.com/formfindregulators.php) were particularly used, and only documented regulations with expression evidence were taken into consideration for all the following analyses. For the analysis of downstream transcription regulatory networks, the significantly differentially expressed genes (SDEGs) identified by RNA-seq analysis were searched for target genes to identify the significantly differentially expressed transcription factors (SDETFs) using the Search for Genes tool, which were further searched for regulatory associations against the SDEGs to identify the given SDETFs and their specifically regulated target SDEGs using the Search for Associations tool. The SDETF-associated SDEGs were then divided into two groups depending on their increased or decreased expression and separately tested for GO biological process enrichment using FunSpec with a *p*-value cutoff of 0.05 and Bonferroni correction (Robinson et al., 2002) (Supplementary Table S5). Depending on whether the SDETFs and SDEGs showed increased or decreased expression in the core TF deletion strains, the regulatory relationship between the core TFs and the SDETFs as well as the SDETFs and enriched GO biological processes of their associated SDEGs were deduced. If the expression level of the downstream SDETF decreased, the core TF acted as an activator to the SDETF, otherwise it acted as an inhibitor. If the downstream SDETF and its associated SDEGs in the enriched GO biological processes showed the same trend of increased or decreased expression, the SDETF would act as an activator of its associated GO biological processes, otherwise acting as an inhibitor if the downstream SDETF and its associated SDEGs showed the opposite trend of expression changes.

To identify the transcription factors upstream of the core TFs and the SDEGs identified in the comparison of the core TF deletion strain versus the wild type strain, on the one hand, the SDEGs were searched against all of the transcription factors in the YEASTRACT database using the TFRank method in the tool of Rank by TF with the default heat diffusion coefficient of 0.25 (Goncalves et al., 2011), allowing us to select and rank transcription factors potentially involved in the regulation of all the SDEGs. On the other hand, using the Search for TFs tool, the core TFs, including Sin3p, Srb2p and Mig1p were also searched for transcription factors that are documented to regulate the transcriptional expression of these core TFs. Eventually, the TFs that are from the top six ranked TFs and from documented TFs regulating the core TFs but not from the downstream SDETFs are targeted to most likely play regulatory roles upstream of the core TFs at a higher level.

### Real Time Quantitative PCR Validation

To confirm how the upstream TFs activated or inhibited *SIN3*, *SRB2*, *MIG1* and their downstream regulated TF *HAC1*, mRNA expression levels of these TFs in the deletion strains of the upstream TFs were measured by using real-time quantitative PCR (qPCR). The culture conditions of these strains were consistent with the conditions of those strains for RNA sequencing. Actin1 gene (*ACT1*) was chosen as endogenous gene. The qPCR primers are listed in Supplementary Table S2. Total RNA was extracted using the RNAsimple Total RNA kit (Tiangen Biotech, Beijing, China) according to the manufacturer’s instructions. The RNA samples were treated with DNase I (TaKaRa Bio, Dalian, China), and 1.0 µg of total RNA was reverse transcribed into cDNA using the PrimeScript RT Reagent kit (TaKaRa). 25 ng of cDNA was used for each qPCR reaction. SYBR Green qPCR was performed using SYBR Premix Ex Taq (TaKaRa) on the Roche LightCycler^®^96 System (Roche Applied Science, Basel, Switzerland) at 95°C for 60 s and 40 cycles at 95°C 15 s and 60°C for 60 s. The data were analyzed using the 2^−ΔΔCt^ method as previously described (Livak and Schmittgen, 2001).

### Analytical Methods and Calculation

Cell growth was detected at OD_600_ using a SpectraMax M2 spectrophotometer (Molecular Devices, United States). Cell cultures or suspensions were appropriately diluted, thereby resulting in visible absorption values in the range from 0.2 to 0.8. Glucose and ethanol concentrations were measured by high performance liquid chromatography (HPLC) on an Agilent 1260 system (Agilent, United States), a refractive index detector and a fast acid column 100 mm length × 7.8 mm internal diameter (RFQ; Phenomenex Inc., Torrance, CA, United States). The column was eluted with 0.01 N H_2_SO_4_ at a flow rate of 0.6 ml/min at 55°C. Fermentation parameters, including the maximum glucose consumption rate (q_s_max) and ethanol productivity (P_EtOH_) were calculated corresponding to the fermentation profiles using Originlab^®^ Origin 8 as previously reported (Lin et al., 2014).

## Results

### Key TF Deletions had Distinct Effects on Fermentation Capacities and Heat-Shock Survival of an Industrial *S. cerevisiae* Strain Under Thermal Stress

**FIGURE 1:**
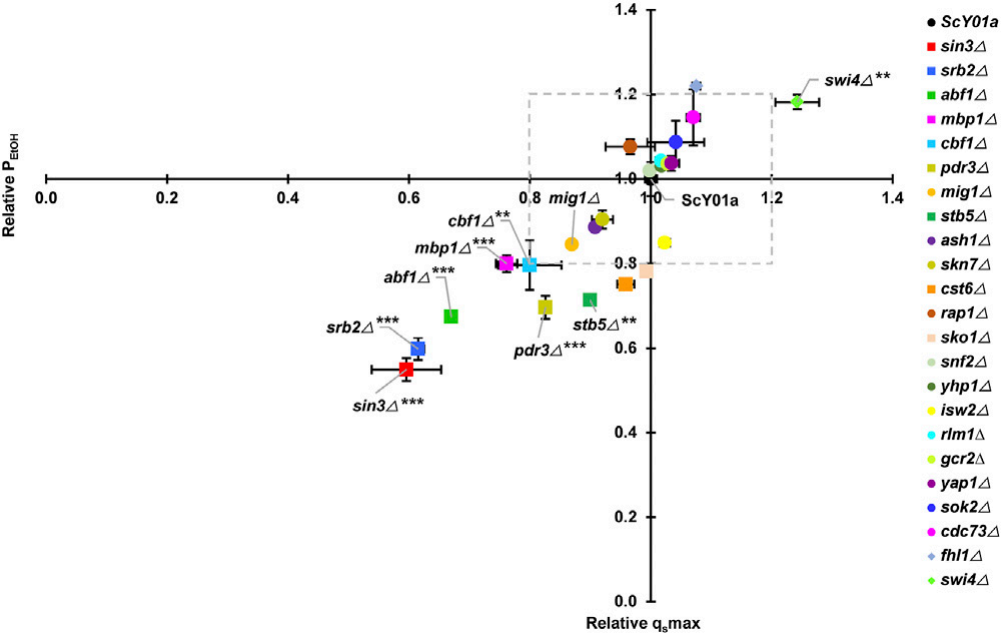
Effects of key TF deletions on fermentation rates at elevated temperature. The relative glucose consumption rate (q_s_max) and ethanol productivity (P_EtOH_) were obtained by comparisons to the wild-type strain ScY01a (normalized to 1.0). Plots of relative q_s_max versus relative P_EtOH_ are shown. The legends from top to bottom are arranged in descending order of relative qsmax at 40°C. Each point represents duplicate fermentations using a starting glucose concentration of 200 g/L and starting OD_600_ of 0.5. The glucose consumption rate and ethanol productivity for the ScY01a strain at 40°C were 10.33 (±0.15) g/L/h and 4.91 (±0.21) g/L/h, and those at 30°C were 13.65 (±2.09) g/L/h and 5.83 (±1.10) g/L/h, respectively. Statistical analysis was performed using two-way ANOVA (with strains and fermentation rate including q_s_max and P_EtOH_ as the factors) followed by Tukey’s multiple-comparison posttest (*p < 0.05, **p < 0.01, ***p < 0.001).

To test whether the twenty-three previously identified TFs specifically associated with long-term thermotolerance affect the high-temperature ethanol fermentation capacities of yeast at the physiological level, we first constructed twenty-three TF single deletion strains, including two knockout strains in our previous report (Xiao et al., 2018). Both the industrial *S. cerevisiae* strain ScY01a and its single TF deletion strains were subjected to fermentation experiments at high temperature (40°C) using normal temperature (30°C) for comparison. This means that yeast cells suffered from a continuous long-term thermal stress of 40°C during the whole course of fermentation for 36 h. The 23 TF deletion mutant strains in this study are listed in Supplementary Table S1. Among these 23 TFs, five TFs *SIN3*, *SRB2, ABF1*, *MBP1* and *CBF1*, seemed to be particularly important for maintaining physiological activities at high temperature because their deletions significantly weakened high-temperature fermentation capacities with more than 20% decreases in fermentation rates including the maximum glucose consumption rate (q_s_max) and ethanol productivity (P_EtOH_) (Figure 1). Meanwhile, except for *SIN3*, deletion of the other four TFs hampered cell growth at high temperature (Supplementary Figure S1A), pointing out their significance in cell proliferation under prolonged thermal stress. In contrast, when fermenting at normal temperature (30°C), deletion of *SRB2* also led to a remarkable decrease in fermentation capacity with an approximately 40% decline in the fermentation rates (Supplementary Figure S2), whereas deletion of *SIN3* or *ABF1* resulted in obvious increases in the fermentation rates, and deletion of *MBP1* or *CBF1* had almost no impact on fermentation rates. Except that *MBP1* deletion seemed to be significantly beneficial to cell growth at normal temperature, the other four TFs had no significant influence on cell growth (Supplementary Figure S1B). Additionally, among the twenty-three TFs, another two TFs, *PDR3* and *STB5*, might also be important for maintaining fermentation capacities at high temperature, because their deletions significantly reduced the maximum glucose consumption rate (q_s_max) with a more than 20% decrease and somehow had a negative effect on the rate of ethanol production (Figure 1). However, cell growth at high temperature was not influenced by deletion of *PDR3* or *STB5* (Supplementary Figure S1A). In contrast, deletion of *PDR3* or *STB5* had no obvious effect on physiological activities and cell proliferation at normal temperature (Supplementary Figure S1B). On the other hand, only *SWI4* deletion was found to be significantly propitious to fermentation capacity at high but not normal temperature and had no effect on cell growth at either high or normal temperature.

**FIGURE 2:**
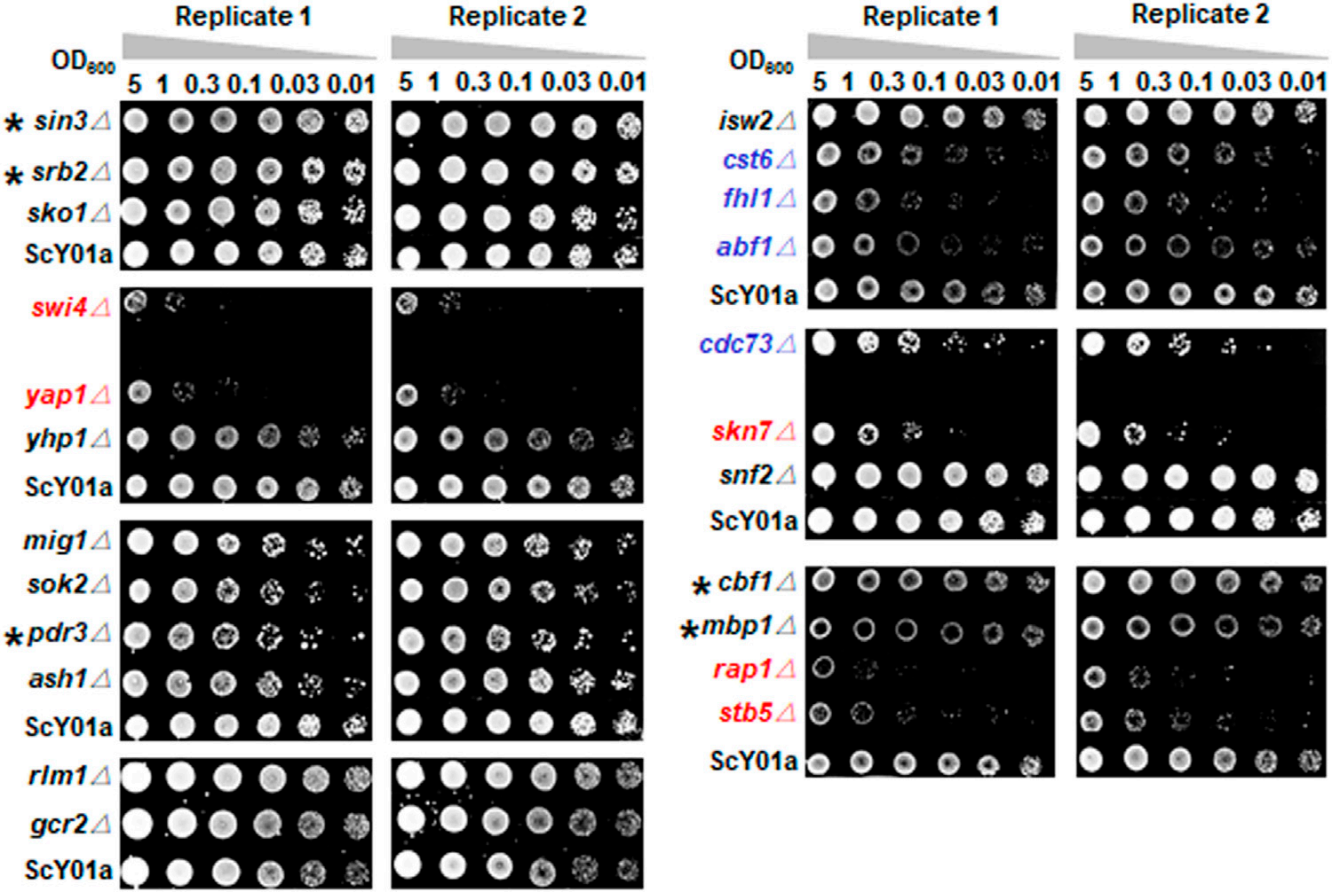
Heat-shock survival test of key TF deletion strains. The 23 TF deletion strains and the wild type strain ScY01a (Supplementary Table S1) were grown in biological duplicates to early-log phase in YP medium containing 200 g/L glucose. Cells were harvested and resuspended to 5.0 OD_600_ and serially diluted to the OD_600_ indicated and spotted on YPD plates after treatment at 50°C for 30 min. The plates were incubated for 2 days at 30°C and imaged. The single deletion strains of five TFs showing severely decreased viabilities in contrast to the wild-type ScY01a are indicated in red. The single deletion strains of four TFs showing decreased viabilities to a relatively lower extent are indicated in blue. Five TFs specifically associated with long-term thermotolerance, whose single deletions showed no effects on heat-shock survival, are labelled with asterisks.

To distinguish TFs specifically associated with long-term thermotolerance from specific TFs of heat shock response for industrial *S. cerevisiae* strain, heat-shock survival was determined for cells of the 23 single TF deletion strains and the wild type strain ScY01a before and after heat shock treatment at 50°C. Cell viabilities showed no obvious differences among the TF deletion and wild-type strains before heat shock treatment (Supplementary Figure S3) but were distinctly influenced after heat shock treatment (Figure 2). Among the 23 TFs, the single deletions of five TFs, *SWI4*, *YAP1*, *SKN7*, *RAP1* and *STB5*, severely decreased cell viability upon heat shock treatment, among which the deletion of *SWI4* and *STB5* showed positive and negative effects on high-temperature fermentation capacities, respectively, and the deletion of the other four TFs showed no effect on high-temperature fermentation capacities (Figure 1). This observation indicated that *SWI4* might play quite different regulatory roles in response to long- and short-term thermal stresses and that *STB5* might be required for maintaining cell activities under both long- and short-term thermal stresses. Additionally, among the 23 TFs, the single deletions of another four TFs, including *CST6*, *FHL1*, *ABF1* and *CDC73*, also resulted in obviously adverse effects on cell viability upon heat shock treatment, among which the deletion of *ABF1* showed negative effects on high-temperature fermentation capacities, and the deletion of the other five TFs showed no effect on high-temperature fermentation capacities (Figure 1). This result suggested that *ABF1* might also be required for maintaining cell activities under both long- and short-term thermal stresses. Combining the results of evaluating the effects of the 23 TF deletions on cell activities under prolonged thermal stress (Figure 1) and short-term heat shock stress (Figure 2), a total of five TFs of the 23 TFs, including *SIN3*, *SRB2*, *MBP1*, *CBF1* and *PDR3*, were verified to be specifically required for long-term thermal stress tolerance because their deletions significantly decreased high-temperature fermentation capacities but had no effects on heat-shock survival.

Taken together, *SIN3* and *SRB2* deletions severely reduced the fermentation capacities of industrial *S. cerevisiae* strains under long-term thermal stress but had no apparent effects on heat-shock survival under short-term thermal stress. This indicated that Srb2p and Sin3p were specific TFs in response to long-term thermal stress. Unexpectedly, the deletion of *MIG1*, which was previously identified to be the most significantly upregulated specific transcription factor at prolonged thermal stress (Xiao et al., 2018), showed no apparent effect on cell growth and physiological activity at prolonged thermal stress or heat shock survival.

### Comparative Transcriptome Profiling Analyses Uncovered Genes and Pathways Regulated by Sin3p, Srb2p or Mig1p Under Prolonged Thermal Stress

Based on comparing the fermentation capacities of key TF deletions and induction levels of transcriptional expression of twenty-three previously identified TFs at prolonged thermal stress (Xiao et al., 2018), Sin3p, Srb2p and Mig1p were considered to be the core TFs specifically in response to long-term thermal stress. To reveal the genes and pathways regulated by the core TFs at prolonged thermal stress and further understand the transcriptional regulatory mechanism of thermotolerance associated with the core TFs, genome-wide transcriptome analysis using RNA-seq technology was conducted for exponential cells of the core TF deletion strains and the wild type strain ScY01a grown at high temperature and normal temperature as a control.

Overall, the expression of 1131 gene was influenced and regulated by core TFs, including Sin3p, Srb2p and Mig1p, under prolonged thermal stress (Supplementary Table S3). Among the 1131 genes, only 49 genes (4.3%) seemed to be regulated by all the core TFs and were enriched in three biological processes including iron ion homeostasis, ion transport and siderophore transport. For specifically regulated genes of each core TF, Sin3p and Srb2p specifically regulated more than 30% of the 1131 genes, respectively. GO analysis showed that the 395 Sin3p-regulated genes (35.0%) were enriched in reciprocal meiotic recombination and meiosis, and the 419 Srb2p-regulated genes (37.0%) were enriched in ribosome biogenesis and rRNA processing. In terms of Mig1p, 67 specifically regulated genes (5.9%) were enriched in the biological process of response to pheromone. In addition, a relatively large proportion of the 1131 genes were regulated by Sin3p and controlled by Srb2p and/or Mig1p (Supplementary Figure S4). Among these genes, 86 genes (7.6%) regulated by both Sin3p and Srb2p were enriched in six biological processes, including metabolic process, amino acid transmembrane transport, carbohydrate metabolic process, transmembrane transport, sporulation resulting in formation of a cellular spore, and amino acid transport, and 60 genes (5.3%) regulated by both Sin3p and Mig1p were enriched in the biological process of glucose transport. Although 55 genes (4.9%) were regulated by both Srb2p and Mig1p, no biological process was enriched. All these observations revealed common and specific target genes and biological processes regulated by the core TFs, thereby helping dissect the underlying regulatory networks of long-term thermotolerance associated with the core TFs.

Additionally, comparative transcriptome analysis also revealed that the core TFs might influence the expression of a distinct set of genes at high temperature in contrast to normal temperature. Thus, Venn diagram analysis was further performed to compare SDEGs due to the core TF deletion at 40°C with those at 30°C (Supplementary Table S3), and expression changes exclusively in response to 40°C were analyzed for GO biological process enrichment. Due to *SIN3* deletion, the SDEGs exclusively exhibiting increased expression at 40°C were found to be enriched in six biological processes, including transmembrane transport, oxidation-reduction process, tricarboxylic acid cycle, transport, ascospore wall assembly, and mitochondrion degradation, while the SDEGs exclusively exhibiting decreased expression at 40°C were enriched in *de novo*’ IMP biosynthetic process (Supplementary Table S4). Notably, the SDEGs showing increased expression at 40°C but decreased expression at 30°C were enriched in glycogen and carbohydrate metabolic processes, while the SDEGs showing decreased expression at 40°C but increased expression at 30°C were enriched in translation and ribosomal small subunit assembly (Supplementary Table S4). These results suggested that Sin3p seemed to have quite different regulatory effects on gene expression at high and normal temperatures. When *SRB2* was deleted, the SDEGs exclusively exhibiting increased expression at 40°C were found to be enriched in amino acid transmembrane transport, while the SDEGs exclusively exhibiting decreased expression at 40°C were enriched in ascospore wall assembly, maltose metabolic process, and sporulation resulting in the formation of a cellular spore (Supplementary Table S4). Additionally, only 11 and 9 SDEGs due to *SRB2* deletion showed opposite expression changes at 40°C and 30°C, and no significant enrichment was observed (Supplementary Table S4). These results suggested that Srb2p might also play an important regulatory function at high temperature, although Srb2p seemed to impact much less gene expression at high temperature than at normal temperature. Upon *MIG1* deletion, interestingly, the SDEGs exclusively exhibiting increased expression at 40°C were found to be enriched in eight biological processes including iron ion homeostasis, siderophore transport, ion transport, transmembrane transport, response to pheromone, ‘*de novo*’ IMP biosynthetic process, purine nucleotide biosynthetic process and metabolic process, while the SDEGs exclusively exhibiting decreased expression at 40°C were enriched in protein folding (Supplementary Table S4). Additionally, only seven SDEGs due to *MIG1* deletion showed opposite expression changes at 40°C and 30°C, and no significant enrichment was observed (Supplementary Table S4). These results suggested that Mig1p might influence a small but specific set of gene expression at high temperature compared to normal temperature.

### Sin3p, Srb2p and Mig1p Regulated Their Transcriptional Regulatory Networks Through Multiple Downstream TFs

**FIGURE 3:**
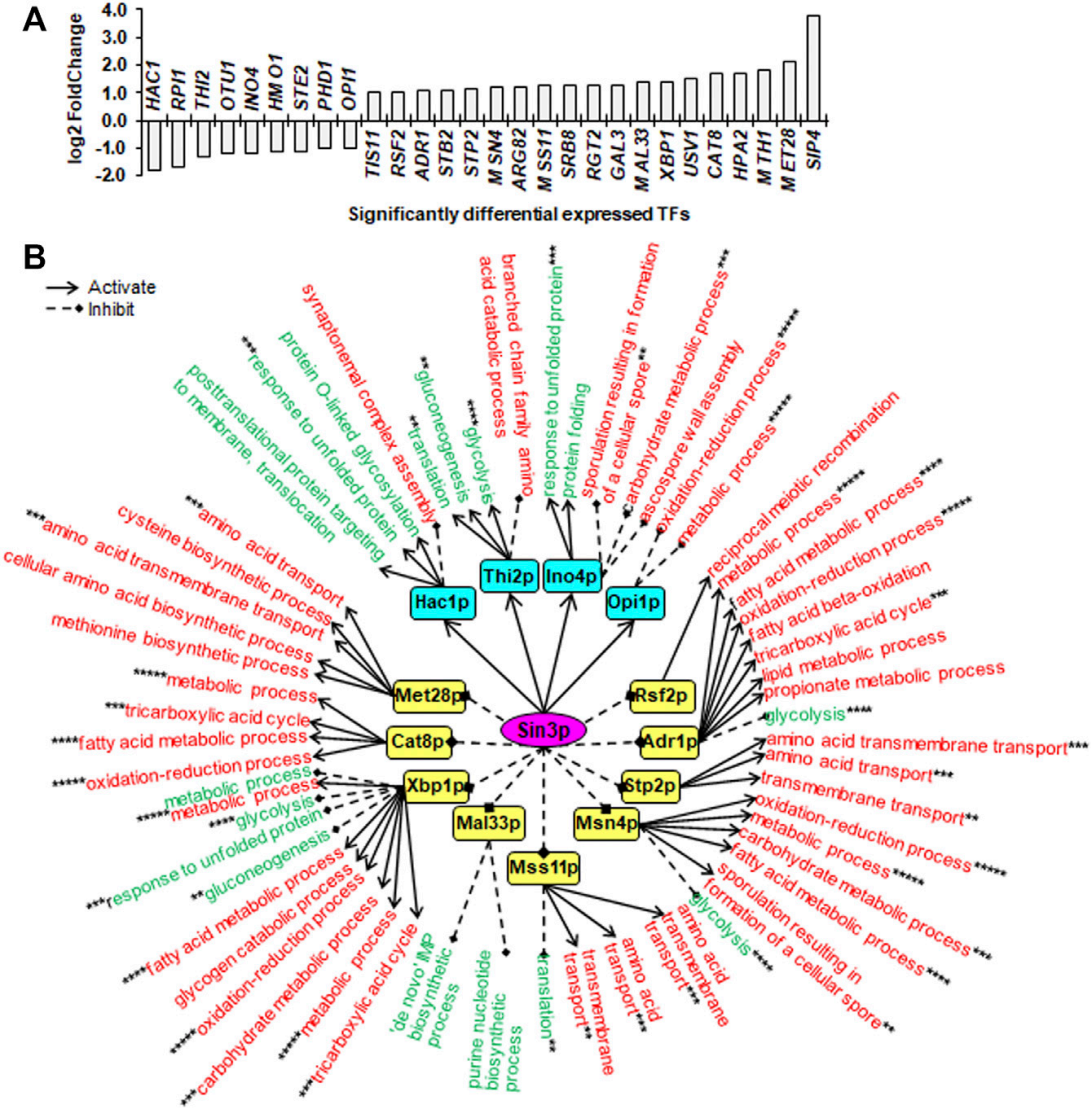
Downstream SDETFs and transcription regulatory network regulated by Sin3p. **(A)** Significantly differentially expressed transcription factors (SDETFs) due to *SIN3* deletion. **(B)** Downstream transcription regulatory networks regulated by Sin3p. Sin3p is placed in the core position of the network and shown in a purple and elliptic box. The middle layer is composed of SDETFs caused by *SIN3* deletion. The SDETFs activated by Sin3p, thus showing decreased expression due to *SIN3* deletion, are shown in aqua boxes. The SDETFs inhibited by Sin3p, thus showing increased expression due to *SIN3* deletion, are shown in yellow boxes. The terminal layer consists of enriched GO biological processes of SDEGs associated with those SDETFs. The GO biological processes, in which the SDETF-associated SDEGs showing increased expression are enriched, are shown in red. The GO biological processes, in which the SDETF-associated SDEGs showing decreased expression are enriched, are shown in green. Solid arrows represent the regulatory relationship of activation. Dashed diamond arrows represent the regulatory relationship of inhibition. The GO biological process regulated by multiple TFs is indicated using asterisks, whose number was equal to the number of TFs.

**FIGURE 4:**
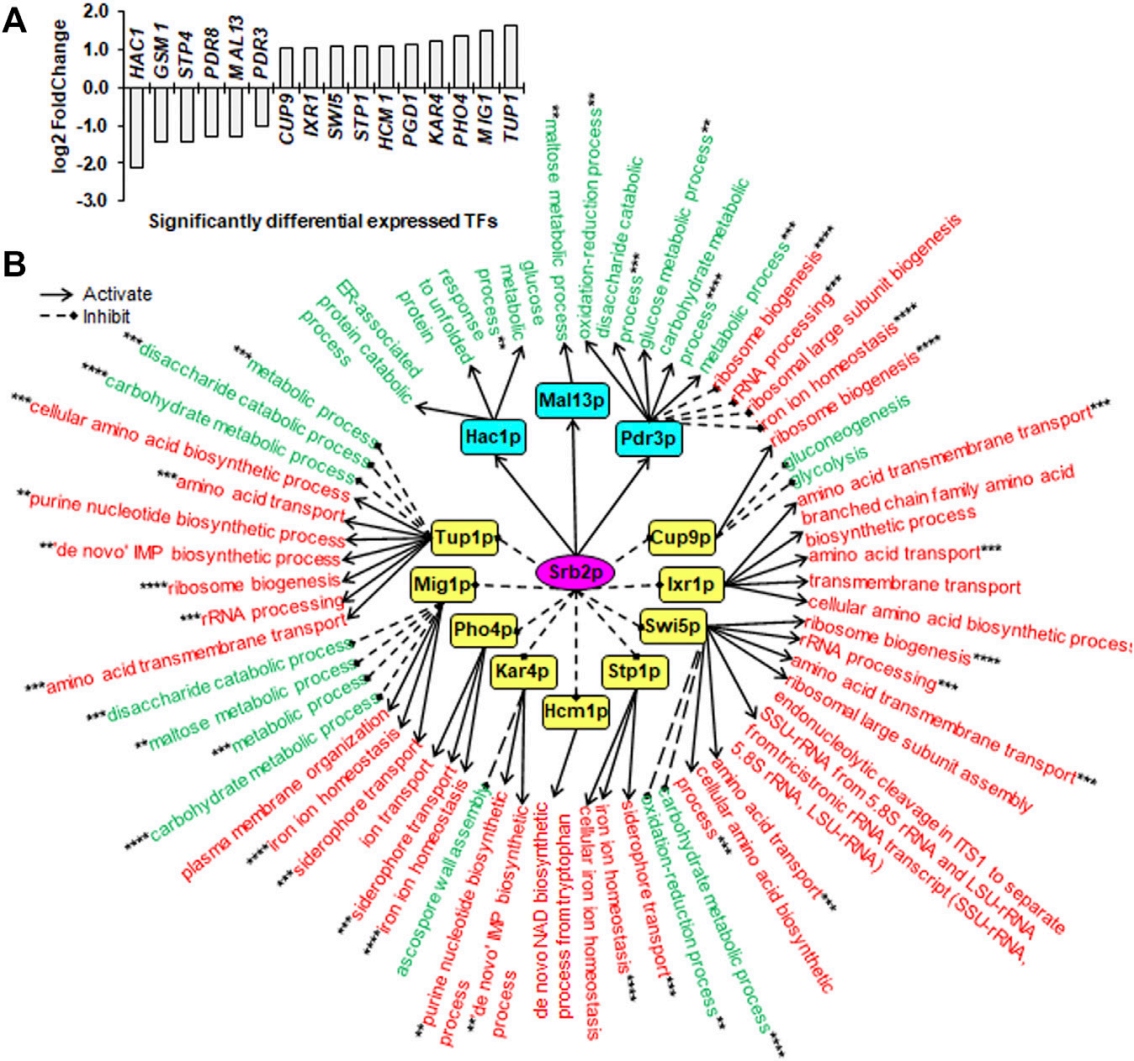
Downstream SDETFs and transcription regulatory network regulated by Srb2p. **(A)** Significantly differentially expressed transcription factors (SDETFs) due to *SRB2* deletion. **(B)** Downstream transcription regulatory networks regulated by Srb2p. Srb2p is placed in the core position of the network and shown in a purple elliptic box. The middle layer is composed of SDETFs caused by *SRB2* deletion. The SDETFs activated by Srb2p, thus showing decreased expression due to *SRB2* deletion, are shown in aqua boxes. The SDETFs inhibited by Srb2p, thus showing increased expression due to *SRB2* deletion, are shown in yellow boxes. The terminal layer consists of enriched GO biological processes of SDEGs associated with those SDETFs. The GO biological processes, in which the SDETF-associated SDEGs showing increased expression are enriched, are shown in red. The GO biological processes, in which the SDETF-associated SDEGs showing decreased expression are enriched, are shown in green. Solid arrows represent the regulatory relationship of activation. Dashed diamond arrows represent the regulatory relationship of inhibition. The GO biological process regulated by multiple TFs is indicated using asterisks, whose number was equal to the number of TFs.

**FIGURE 5:**
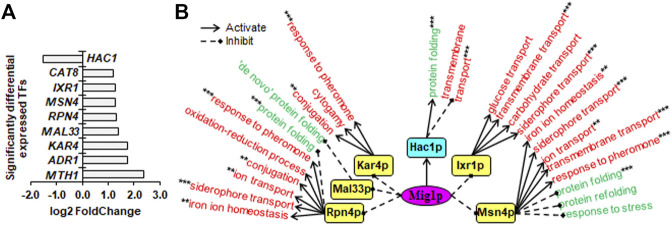
Downstream SDETFs and transcription regulatory network regulated by Mig1p. **(A)** Significantly differentially expressed transcription factors (SDETFs) due to *MIG1* deletion. **(B)** Downstream transcription regulatory networks regulated by Mig1p. Mig1p is placed in the core position of the network and shown in a purple and elliptic box. The middle layer is composed of SDETFs caused by *MIG1* deletion. The SDETFs activated by Mig1p, thus showing decreased expression due to *MIG1* deletion, are shown in aqua boxes. The SDETFs inhibited by Mig1p, thus showing increased expression due to *MIG1* deletion, are shown in yellow boxes. The terminal layer consists of enriched GO biological processes of SDEGs associated with those SDETFs. The GO biological processes, in which the SDETF-associated SDEGs showing increased expression are enriched in, are shown in red. The GO biological processes, in which the SDETF-associated SDEGs showing decreased expression are enriched, are shown in green. Solid arrows represent the regulatory relationship of activation. Dashed diamond arrows represent the regulatory relationship of inhibition. The GO biological process regulated by multiple TFs is indicated using asterisks, whose number was equal to the number of TFs.

To unveil downstream transcription regulatory networks regulated by core TFs including Sin3p, Srb2p and Mig1p, we identified significantly differentially expressed TFs (SDETFs) due to core TF deletions (Figures 3A, 4A, 5A) and their associated SDEGs (Supplementary Table S5) using the YEASTRACT database (Teixeira et al., 2006; Teixeira et al., 2014). We then performed GO enrichment analysis for the SDEGs associated with the SDETFs and further analyzed the regulatory relationship between the core TFs and the SDETFs as well as the SDETFs and the enriched GO biological processes of their associated SDEGs (Supplementary Table S5). For each regulatory network centered on the core TF, the core TF was placed in the central position. The middle layer was composed of SDETFs caused by the core TF deletion. The terminal layer consisted of enriched GO biological processes of SDEGs associated with those SDETFs. Then, the regulatory relationships between layers were deduced depending on the fact that the SDETFs and SDEGs showed increased or decreased expression (Figures 3B, 4B, 5B). Thus, how each core TF was involved in regulating gene expression and biological process activities could be clearly dissected. Additionally, to focus on the transcription regulatory networks in response to high-temperature fermentation, only the dissection results of SDEGs at high temperature (40°C) were described in detail, while the downstream TFs as well as their associated SDEGs and enriched GO biological processes at normal temperature (30°C) were also included in Supplementary Table S5 for a reference.

Due to *SIN3* deletion, nine TFs showed decreased expression, and 19 TFs showed increased expression (Figure 3A). The dissected Sin3p-regulated transcriptional network covered 13 downstream TFs as well as 89.8% of upregulated SDEGs and 91.3% of downregulated SDEGs (Figure 3B). By activating Hac1p, Thi2p, Ino4p and Opi1p, Sin3p might positively regulate transcription of SDEGs enriched in seven biological processes, but negatively regulate the transcription of SDEGs enriched in seven biological processes. On the other hand, by inhibiting eight TFs, including Rsf2p, Adr1p, Stp2p, Msn4p, Mss11p, Xbp1p, Cat8p and Met28p, Sin3p might negatively regulate the transcription of SDEGs enriched in 17 biological processes. Additionally, by repressing five TFs, including Adr1p, Msn4p, Mss11p, Mal33p and Xbp1p, Sin3p positively regulated the transcription of SDEGs enriched in seven biological processes.

Due to *SRB2* deletion, six TFs showed decreased expression, and 10 TFs showed increased expression (Figure 4A). The dissected Srb2p-regulated transcriptional network covered 12 downstream TFs as well as 92.4% of upregulated SDEGs and 87.2% of downregulated SDEGs (Figure 4B). By activating Hac1p, Mal13p and Pdr3p, Srb2p might positively regulate the transcription of SDEGs enriched in eight biological processes. Additionally, by inducing Pdr3p, Srb2p might negatively regulate the transcription of SDEGs enriched in four biological processes. On the other hand, by inhibiting 10 TFs, including Cup9p, Ixr1p, Swi5p, Stp1p, Hcm1p, Kar4p, Pho4p, Mig1p and Tup1p, Srb2p might negatively regulate the transcription of SDEGs enriched in 17 biological processes. Additionally, by repressing five TFs including Cup9p, Swi5p, Kar4p, Mig1p and Tup1p, Srb2p positively regulated the transcription of SDEGs enriched in eight biological processes.

Due to *MIG1* deletion, only one TF, Hac1p, showed decreased expression, and eight TFs showed increased expression (Figure 5A). The dissected Mig1p-regulated transcriptional network covered six downstream TFs as well as 92.7% of upregulated SDEGs and 93.8% of downregulated SDEGs (Figure 5B). By activating Hac1p, Mig1p might positively regulate transcription of SDEGs enriched in the biological process of protein folding, while negatively regulate the transcription of SDEGs enriched in the biological process of transmembrane transport. On the other hand, by inhibiting Ixr1p, Msn4p, Rpn4p and Kar4p, Mig1p negatively regulated the transcription of SDEGs enriched in eight biological processes. Additionally, by repressing Msn4p, Rpn4p and Mal33p, Mig1p might positively the regulate transcription of SDEGs enriched in four biological processes.

Remarkably, in all three Sin3p-, Srb2p- and Mig1p-regulated networks, many SDEGs and their enriched biological processes were found to be regulated by multiple TFs. For instance, the biological process of response to unfolded protein, which showed decreased gene expression due to *SIN3* deletion, was positively regulated by two Sin3p-activated TFs, Hac1p and Ino4p, and negatively regulated by one Sin3p-inhibited TF, Xbp1p (Figure 3B). Furthermore, most TFs regulated by Sin3p, Srb2p and Mig1p could function as activators or inhibitors for different genes enriched in different biological processes, enhancing the complexity of transcriptional regulatory networks.

### Five Potential Upstream TFs Were Involved in Regulating Sin3p, Srb2p and Mig1p as Well as Their Regulated Genes

**FIGURE 6:**
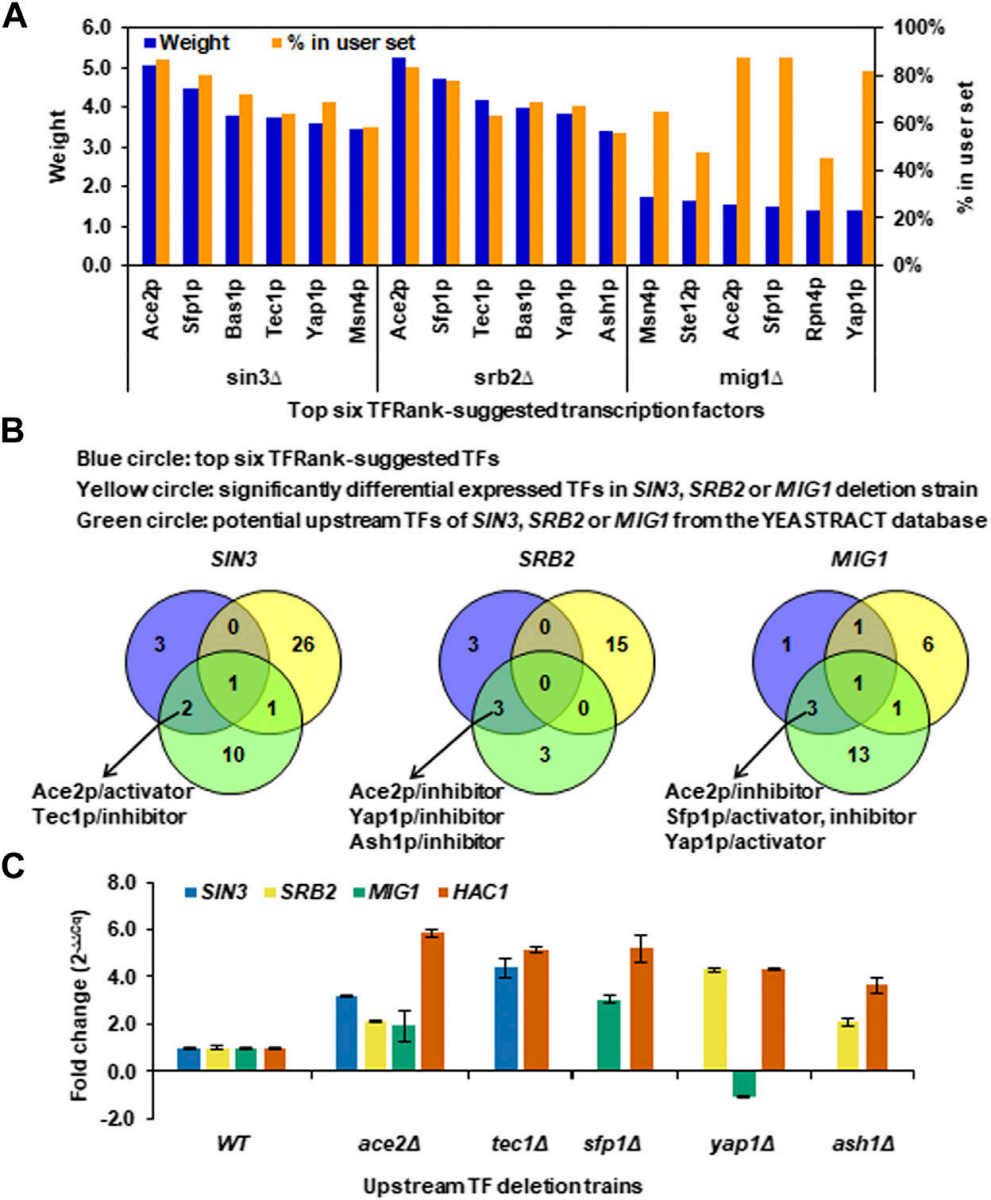
Upstream TFs regulating the core TFs and their downstream regulated genes under long-term thermal stress. **(A)** Top six TFRank-suggested transcription factors using the tool of Rank by TF in the YEASTRACT database and all the SDEGs caused by *SIN3*, *SRB2* or *MIG1* deletion as target genes. Regulation weights were given by the TFRank analysis. The percentage of target genes in the user set was calculated as the ratio of the number of SDEGs the TF can regulate to the number of total SDEGs. **(B)** Venn diagram analysis for determining the TFs that most likely play regulatory roles upstream of the core TFs at a higher level. **(C)** Transcriptional changes of *SIN3*, *SRB2*, *MIG1* and *HAC1* in the *ace2Δ*, *tec1Δ*, *sfp1Δ*, *yap1Δ* and *ash1Δ* deletion strains. Data represent the mean and standard error of triplicate cultures (n = 3) for each strain.

To further deduce potential TFs involved in regulating Sin3p, Srb2p and Mig1p as well as their regulated genes, we explored the TFRank method in the YEASTRACT database, which was developed to select and prioritize the relevant regulatory players for a list of genes of interest (Goncalves et al., 2011). All the SDEGs of each core TF deletion were used to rank potential TFs, and the top six TFs corresponding to the highest regulation weight and separately regulating more than 40% of SDEGs were considered to be the most relevant mediators of the yeast transcriptional response to the core TF deletion (Figure 6A). The top six TFRank-suggested TFs for the SDEGs of *SIN3* or *SRB2* deletion showed higher weights than those of *MIG1* deletion (Figure 6A). Since the TFRank method takes into account integrated rather than isolated transcriptional control, while walking through the global TF network, the regulation weight also indicated the significance of the top six ranked TFs and their associated target genes in the whole regulatory network. Therefore, the top six ranked TFs and their targets of the Sin3p- and Srb2p-regulated networks might play major roles in the global regulatory network in response to long-term thermal stress, and those of the Mig1p-regulated network might play a moderate role.

To more explicitly predict the TFs that most likely play regulatory roles upstream of the core TFs, the SDETFs whose expression levels were influenced by the core TF deletion were first eliminated from the top six TFs to avoid disturbance from the downstream layer of the regulatory network (Figure 6B). Furthermore, only those of the top six TFs, which were also found to be the potential upstream TFs regulating the core TF according to documented expression evidence in the YEASTRACT database, were deemed to be most likely involved in regulating the core TF as well as their regulated genes (Supplementary Table S6, Figure 6B). Eventually, Ace2p was deduced to be an activator of *SIN3*, but an inhibitor of *SRB2* and *MIG1* (Figure 6B). Yap1p is an inhibitor of *SRB2* but an activator of *MIG1*. Tec1p, Ash1p and Sfp1p were specific TFs for *SIN3*, *SRB2* and *MIG1*, respectively (Figure 6B).

To experimentally confirm the regulatory roles of the predicted upstream regulators, changes in the mRNA levels of *SIN3*, *SRB2* and *MIG1* as well as their common regulated target *HAC1* in the comparison of the upstream TF deletion strains versus the wild type strain were detected using real-time quantitative PCR (Figure 6C). *ACE2* deletion resulted in significantly increased transcription of *SIN3* (fold change >2), indicating that Ace2p is an inhibitor of *SIN3* instead of an activator documented at YEASTRACT. *ACE2* deletion also resulted in increased transcription of *SRB2* and *MIG1*, confirming the inhibitory regulation of Ace2p, although the degrees of transcriptional changes were not as significant as that of *SIN3*. The upstream regulators, Tec1p, Sfp1p and Ash1p, were confirmed to be inhibitors of *SIN3*, *MIG1* and *SRB2*, respectively, due to increased transcription of the TFs upon upstream regulator deletion. Yap1p, as an inhibitor, was confirmed for *SRB2* but not for *MIG1*, since the fold changes in the transcription of *SRB2* and *MIG1* upon *ACE2* deletion were 4.3 and -1.1, respectively.

### A Combined Hierarchical Transcriptional Regulatory Network Centered on Sin3p, Srb2p and Mig1p Was Dissected to Be Specifically Involved in the Response to Long-Term Thermal Stress

**FIGURE 7:**
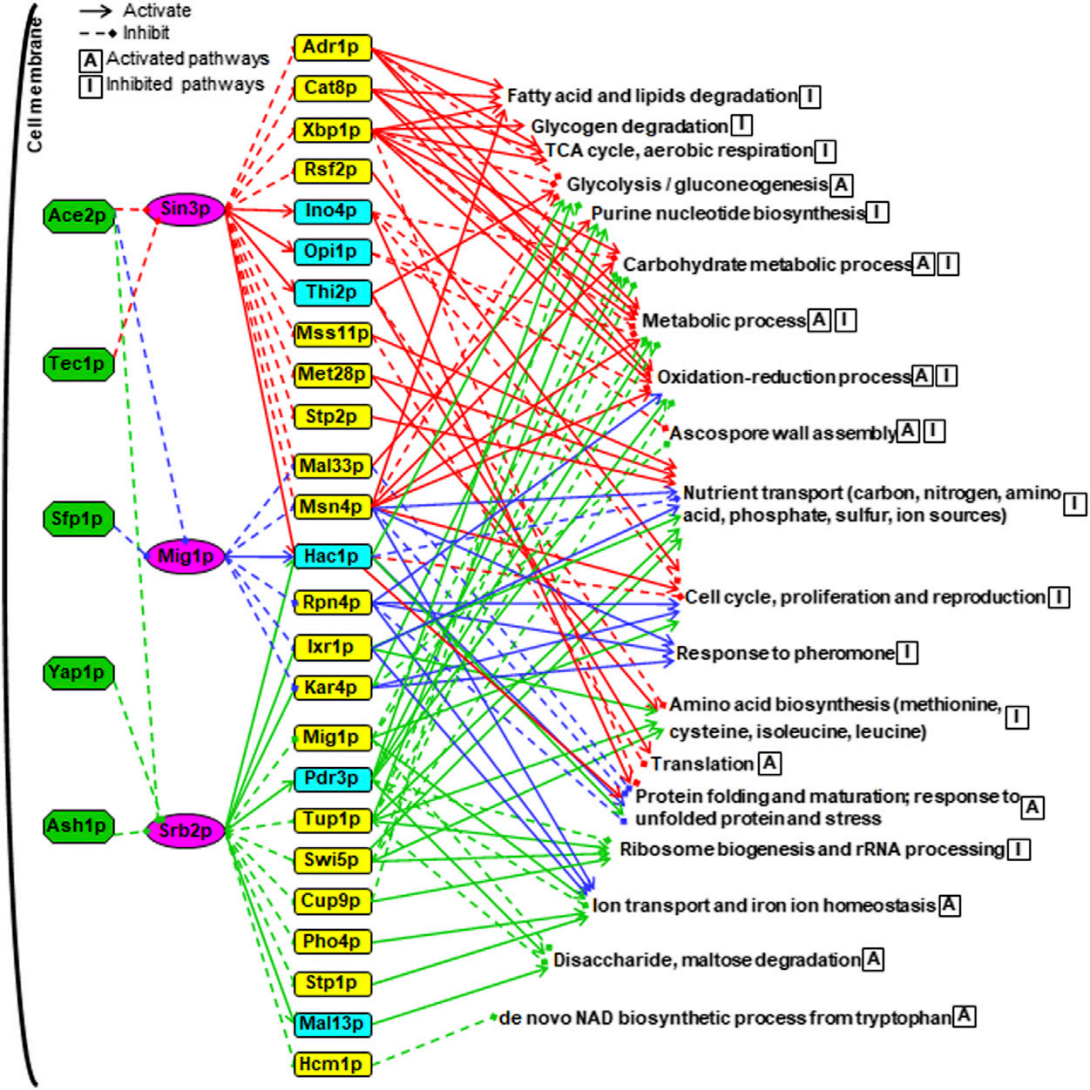
A combined hierarchical transcriptional regulatory network centered on Sin3p, Srb2p and Mig1p. The upstream TFs were shown in green boxes. The core TFs were shown in purple boxes. The SDETFs activated by the core TFs, thus showing decreased expression due to the core TF deletion, are shown in aqua boxes. The SDETFs inhibited by the core TFs, thus showing increased expression due to the core TF deletion, are shown in yellow boxes. The enriched GO biological processes of SDEGs activated by the core TFs, thus showing decreased expression due to the core TF deletion, are indicated with the letter A in a box. The enriched GO biological processes of SDEGs inhibited by the core TFs, thus showing increased expression due to the core TF deletion, are indicated with the letter I in a box. The components in the Sin3-involved regulatory network are linked by red lines, blue for Mig1p and green for Srb2p. Solid arrows represent the regulatory relationship of activation. Dashed diamond arrows represent the regulatory relationship of inhibition.

To take a clear and comprehensive look at the global transcriptional regulatory network centered on the core TFs, which might contain key players in response to long-term thermal stress, we generated a three-layer regulatory network by combining all the analyses of downstream and upstream TFs and TF-gene associations for the SDEGs due to the core TF deletions (Figure 7, Supplementary Table S7). As apparently indicated, Ace2p seemed to play a general role in the regulation of the whole network by inhibiting Sin3p, Mig1p and Srb2p. In contrast, Tec1p and Sfp1p seemed to play a specific regulatory role by inhibiting Sin3p or by inhibiting Mig1p, respectively. In addition to Ace2p, Yap1p and Ash1p were also involved in inhibiting Srb2p.

The middle layer was composed of 25 TFs, which showed significantly differential expression due to at least one of the core TF deletions (Figure 7). These 25 TFs have been reported to regulate a broad spectrum of target genes involved in different physiological activities of yeast. For instance, Adr1p, Cat8p and Mig1p are in a transcriptional regulatory cascade, that is controlled by the upstream protein kinase Snf1p and involved in regulating glucose repression, nonfermentable carbon utilization, respiration, etc. (Turcotte et al., 2010). Interestingly, Snf1 is also a key player in the response to cellular stress in yeast, such as nutrient limitation, salt stress and heat shock (Sanz, 2003). Additionally, Xbp1p, Rsf2p, Msn4p, Hac1p, Rpn4p, Pdr3p, Cup9p and Hcm1p have also been reported to play regulatory roles in the cellular response to several forms of stress (*Saccharomyces* Genome database, SGD, www.yeastgenome.org). The remaining 25 TFs have been reported to regulate sulfur metabolism, proteasome, invasive growth, phosphate metabolism, etc. (SGD). These results indicated that Sin3p, Srb2p and Mig1p might allow yeast cells to maintain physiological activities under long-term thermal stress by regulating some TFs in the middle layer. Notably, all three core TFs were involved in activating Hac1p expression. Hac1p is an essential TF under conditions that trigger the unfolded protein response (UPR) (Kaufman, 1999), such as heat shock stress. This suggested that Sin3p, Srb2p and Mig1p might be involved in the long-term thermal stress-induced UPR by activating Hac1p.

From the viewpoint of the terminal layer in the whole network, the regulation of the biological processes was fulfilled by collaborating multiple TFs through multiple regulation routes (Figure 7). Among the 19 groups of GO enriched biological processes regulated by the whole network, nutrient transport, cell cycle, proliferation and reproduction were inhibited by three core TF-regulated networks: Sin3p, Mig1p and Srb2p. The oxidation-reduction process containing various pathways was inhibited by the Sin3p-, and Mig1p-regulated networks but activated by the Srb2p-regulated network. Remarkably, the regulatory functions of Sin3p, Mig1p and Srb2p seemed to be well organized to control protein metabolism. Specifically, protein precursor amino acid biosynthesis was inhibited by the Sin3p- and Srb2p-regulated networks. Ribosome biogenesis and rRNA processing were only inhibited by the Srb2p-regulated network, whereas the translation process was only activated by the Sin3p-regulated network. By activating the same TF, Hac1p, all three core TFs-regulated networks were involved in activating protein folding and maturation as well as the response to unfolded proteins and stress.

Some biological processes were regulated by two core TF-regulated networks. The glycolysis and gluconeogenesis pathways were activated by both the Sin3p- and Srb2p-regulated networks, while purine nucleotide biosynthesis was inhibited. Furthermore, the biological processes of carbohydrate metabolic process and metabolic process as well as ascospore wall assembly were inhibited by the Sin3p-regulated networks but activated by the Srb2p-regulated networks. On the other hand, ion transport and iron ion homeostasis were activated by Mig1p- and Srb2p-regulated networks.

Additionally, some biological bioprocesses were specifically regulated by one core TF-regulated network. The biological processes of fatty acid and lipid degradation, glycogen degradation, tricarboxylic acid (TCA) cycle and aerobic respiration were only inhibited by the Sin3p-regulated network. The biological processes activated only by the Srb2p-regulated network were related to disaccharide and maltose degradation as well as *de novo* NAD biosynthetic process from tryptophan. The biological process of the response to pheromone was found to be inhibited only by the Mig1p-regulated network.

## Discussion

Transcriptional regulation plays a pivotal role in yeast defense and adaptation against environmental stresses. Transcriptional control of the short-term heat shock response has been intensively investigated (Gasch et al., 2000; Verghese et al., 2012). In contrast, the mechanism underlying the response to long-term thermal stress has recently attracted considerable attention on the behalf of industrial application of yeast thermotolerance in high temperature fermentation processes (Abdel-Banat et al., 2010; Caspeta et al., 2014; Shui et al., 2015; Lahtvee et al., 2016). In this study, we identified three core TFs specifically involved in regulating the response to long-term thermal stress, including Sin3p, Srb2p and Mig1p. The deletion of *SIN3* and *SRB2* resulted in dramatically decreased fermentation capacities at high temperature but had no effect on cell survival after heat shock treatment (Figures 1, 2). Additionally, *MIG1* was previously observed to be the most significantly upregulated specific TF under prolonged thermal stress (Xiao et al., 2018), although its deletion had no apparent effect on fermentation capacity at high temperature (Figure 1). Based on the analyses of comparative transcriptome profiling of the core TF deletions and transcription regulatory associations, a hierarchical transcriptional regulatory network centered on these three TFs was further generated and dissected, thus providing a better understanding of the regulatory mechanism behind long-term thermal stress tolerance as well as potential targets for transcriptome engineering to improve yeast thermotolerance.

Compared with short-term heat shock, which causes yeast cells to experience transient and severe heat stress thereby causing thermal damage to threaten cell survival, long-term thermal stress is prolonged and moderate, which leads to inhibited cell growth and reduced fermentation capacity. Coincident with distinct physiological effects, transcriptional reprogrammings are also different between the responses to short- and long-term thermal stress. The heat shock response is appropriately considered to prevent severe thermal damage rather than to promote recovery from an existing insult (Verghese et al., 2012). Hsf1p acts as a primary modulator to activate a battery of cytoprotective genes encoding heat shock proteins, Msn2p and Msn4p govern gene expression involved in the general stress response, and another four heat shock TFs, Sfp1p, Pdr3p, Rpn4p and Stp1p regulate genes encoding ribosomal components, proteasomal proteins, RNA-processing factors, and other progrowth proteins, resulting in the repression of the protein biosynthetic capacity (Wu and Chen, 2009; Sakurai and Ota, 2011). In contrast, the response to long-term thermal stress seemed to induce recovery pathways from thermal damage rather than cytoprotective pathways. Long-term high temperature induced transcriptional expression of core TFs, including Sin3p, Srb2p and Mig1p (Xiao et al., 2018). By activating the whole transcription regulatory network centered on the core TFs, only several heat shock proteins were induced (Supplementary Table S3). Although the biological processes of amino acid biosynthesis, ribosome biogenesis and rRNA processing were inhibited, the biological processes of translation, protein folding and maturation were activated (Figure 7). This suggested that yeast cells under prolonged thermal stress could maintain their protein biosynthetic capacity, which is repressed by short-term heat shock. Remarkably, Hac1, a key TF involved in regulating the unfolded protein response, was positively regulated by three core TFs, Sin3p, Srb2p and Mig1p (Figure 7), thus efficiently buffering endoplasmic reticulum stress caused by high temperature. Furthermore, high temperature was previously observed to increase protein turnover, which increases ATP demand for cellular maintenance, leading to the onset of respirofermentative metabolism (Lahtvee et al., 2016). Similarly, glycolysis was positively regulated by the core TF-mediated transcription regulatory network, while the TCA cycle and aerobic respiration were inhibited.

Notably, the core TFs might be endowed with novel regulatory functions at prolonged thermal stress in contrast to normal and short-term thermal stress conditions. Sin3p and Srb2p influenced the expression of many differentially expressed genes involved in translation at normal temperature (30°C) but not at high temperature (40°C) (Supplementary Tables S3, S4). Sin3p, a component of both the Rpd3S and Rpd3L histone deacetylase complexes, is involved in transcriptional repression and activation of diverse processes (Silverstein and Ekwall, 2005). Sin3p positively regulates transcription from the RNA polymerase II promoter in response to heat stress, ensuring cell survival and growth during the temperature increase (Ruiz-Roig et al., 2010). In this study, *SIN3* deletion resulted in severely decreased fermentation capacity at high temperature, confirming its importance for cellular maintenance under thermal stress (Figure 1). Interestingly, three TFs, Adr1, Cat8 and Mth1, which are negatively regulated by the key TF Mig1p in glucose repression (Carlson, 1999; Westholm et al., 2008), showed increased gene expression (Figure 3A). This result indicated that Sin3p negatively regulated these three TFs. Dissection of transcription regulatory associations suggested that gene expression in the TCA cycle and aerobic respiration might be inhibited by Sin3p through Adr1p and Cat8p (Figures 3B, 7). Although Adr1p, Cat8p and Mth1p also showed increased gene expression due to *MIG1* deletion, no enriched biological processes associated with Adr1p and Cat8p were found (Figure 5). Nevertheless, Mig1p was discovered to be involved in regulating stress response TFs and biological processes (Figure 5). For instance, Mig1p could activate Hac1, thus promoting protein folding. Srb2p (Med20), a head module subunit of the RNA pol II mediator complex, was previously reported to play a regulatory role in the repression of RP gene transcription under a wide variety of environmental stresses (Willis et al., 2008). In addition to inhibiting ribosome biogenesis and rRNA processing, Srb2p was also involved in activating glycolysis, ion transport and iron ion homeostasis (Figures 4B, 7). The significance of these altered biological processes in response to prolonged thermal tolerance remains to be further investigated. Additionally, Sin3p, Srb2p and Mig1p were found to be conserved from yeast to fungi or even humans (Ronne, 1995; Boube et al., 2002; Nishida, 2009). However, their roles in the stress response are less known. Thus, this work could provide clues for investigating the regulation of the cell stress response via Sin3p, Srb2p and Mig1p in organisms other than *S. cerevisiae*.

Transcription factors are regarded as desirable targets for manipulating transcriptional states to enhance stress tolerance. In our previously reported study (Xiao et al., 2018), the overexpression of *MIG1* and *SRB2* under their original promoters showed significantly increased fermentation capacities at high temperature. However, when driven by constitutively strong promoters using a high-copy plasmid, the overexpression of *MIG1* and *SRB2* had few or even adverse effects on heat-stressed growth. Similarly, in this study, *SIN3* overexpression under its original promoter slightly enhanced glucose consumption and ethanol production at high temperature, while its overexpression under the strong promoter *TEF1* obviously hampered thermotolerant fermentation (Supplementary Figure S5). All these results suggested that the core TFs Sin3p, Srb2p and Mig1p required for long-term thermotolerance might be dosage-sensitive and subtly regulated to maintain the physiological activities of *S. cerevisiae* cells at high temperature. Overexpression of Ace2p and/or Spf1p was recently reported to be beneficial to resistance to acetic acid and furfural (Chen et al., 2016). While not tested in this study, appropriate perturbation of predicted upstream TFs, including Ace2p, Tec1p, Spf1p, Yap1p and Ash1p, in the whole transcription regulatory network centered on the core TFs could potentially render beneficial regulatory traits in transcription to improve yeast thermotolerance. In the future, more efforts would be worthwhile to develop multiplex fine-tuning approaches to manipulate these TFs, thus improving the long-term thermal stress tolerance of yeast.

## Data Availability

The datasets presented in this study can be found in online repositories. The names of the repository/repositories and accession number(s) can be found in the article/Supplementary Material.
