# Supplementary material for: A Hierarchical Transcriptional Regulatory Network Required for Long-Term Thermal Stress Tolerance in an Industrial Saccharomyces cerevisiae Strain
Source: Front Bioeng Biotechnol. 2022 Jan 18;9:826238. doi: 10.3389/fbioe.2021.826238 (PMC8804346; doi:10.3389/fbioe.2021.826238)
Supplement: Supplementary file 3 [file Table1.DOCX]

Supplementary Material

**Supplementary Table S1. *S. cerevisiae* strains used in this study**

| Strain or Plasmid | Description | Reference or source |
| --- | --- | --- |
| *S. cerevisiae* strains |  |  |
| BY4743 deletion collection | The deletion strain collection of heterozygous diploid strain BY4743 (*MAT*a/α *his3*Δ*1/his3*Δ*1 leu2*Δ*0/leu2*Δ*0 LYS2/lys2*Δ*0 met15*Δ*0/MET15 ura3*Δ*0/ura3*Δ*0*) | EUROSCARF, Frankfurt, Germany |
| ScY01 | Evolved thermotolerant strain from ScY, diploid | (Shui et al., 2015) |
| ScY01a | *MATa* haploid strain derived from ScY01 | (Xiao et al., 2018) |
| ScY01a (ura3∆) | ScY01a, MATa, ura3::KanMX; uracil auxotrophic | (Xiao et al., 2018) |
| ScY01a (*abf1∆*) | ScY01a, *MATa*, *abf1*::KanMX | This study |
| ScY01a (*ash1∆*) | ScY01a, *MATa*, *ash1*::KanMX | This study |
| ScY01a (*cbf1∆*) | ScY01a, *MATa*, *cbf1*::KanMX | This study |
| ScY01a (*cdc73∆*) | ScY01a, *MATa*, *cdc73*::KanMX | This study |
| ScY01a (*cst6∆*) | ScY01a, *MATa*, *cst6*::KanMX | This study |
| ScY01a (*fhl1∆*) | ScY01a, *MATa*, *fhl1*::KanMX | This study |
| ScY01a (*gcr2∆*) | ScY01a, *MATa*, *gcr2*::KanMX | This study |
| ScY01a (*isw2∆*) | ScY01a, *MATa*, *isw2*::KanMX | This study |
| ScY01a (*mbp1∆*) | ScY01a, *MATa*, *mbp1*::KanMX | This study |
| ScY01a (*mig1∆*) | ScY01a, *MATa*, *mig1*::KanMX | (Xiao et al., 2018) |
| ScY01a (*pdr3∆*) | ScY01a, *MATa*, *pdr3*::KanMX | This study |
| ScY01a (*rap1∆*) | ScY01a, *MATa*, *rap1*::KanMX | This study |
| ScY01a (rlm1*∆*) | ScY01a, *MATa*, *rlm1*::KanMX | This study |
| ScY01a (*sin3∆*) | ScY01a, *MATa*, *sin3*::KanMX | This study |
| ScY01a (*sko1∆*) | ScY01a, *MATa*, *sko1*::KanMX | This study |
| ScY01a (*srb2∆*) | ScY01a, *MATa*, *srb2*::KanMX | (Xiao et al., 2018) |
| ScY01a (*stb5∆*) | ScY01a, *MATa*, *stb5*::KanMX | This study |
| ScY01a (*swi4∆*) | ScY01a, *MATa*, *swi4*::KanMX | This study |
| ScY01a (*yhp1∆*) | ScY01a, *MATa*, *yhp1*::KanMX | This study |
| ScY01a (*skn7∆*) | ScY01a, *MATa*, *skn7-(641-672)*::KanMX | This study |
| ScY01a (*snf2 ∆*) | ScY01a, *MATa*, *snf2-(601-631)*:KanMX | This study |
| ScY01a (*sok2∆*) | ScY01a, *MATa*, *sok2-(501-542)*::KanMX | This study |
| ScY01a (*yap1∆*) | ScY01a, *MATa*, *yap1-(631-671)*::KanMX | This study |
| ScY01a (*ace2∆*) | ScY01a, *MATa*, *ace2*::KanMX | This study |
| ScY01a (*tec1∆*) | ScY01a, *MATa*, *tec1*::KanMX | This study |
| ScY01a (*sfp1∆*) | ScY01a, *MATa*, *sfp1*::KanMX | This study |
