# Supplementary material for: A Hierarchical Transcriptional Regulatory Network Required for Long-Term Thermal Stress Tolerance in an Industrial Saccharomyces cerevisiae Strain
Source: Front Bioeng Biotechnol. 2022 Jan 18;9:826238. doi: 10.3389/fbioe.2021.826238 (PMC8804346; doi:10.3389/fbioe.2021.826238)
Supplement: Supplementary file 4 [file Presentation1.PDF]

Supplementary Material

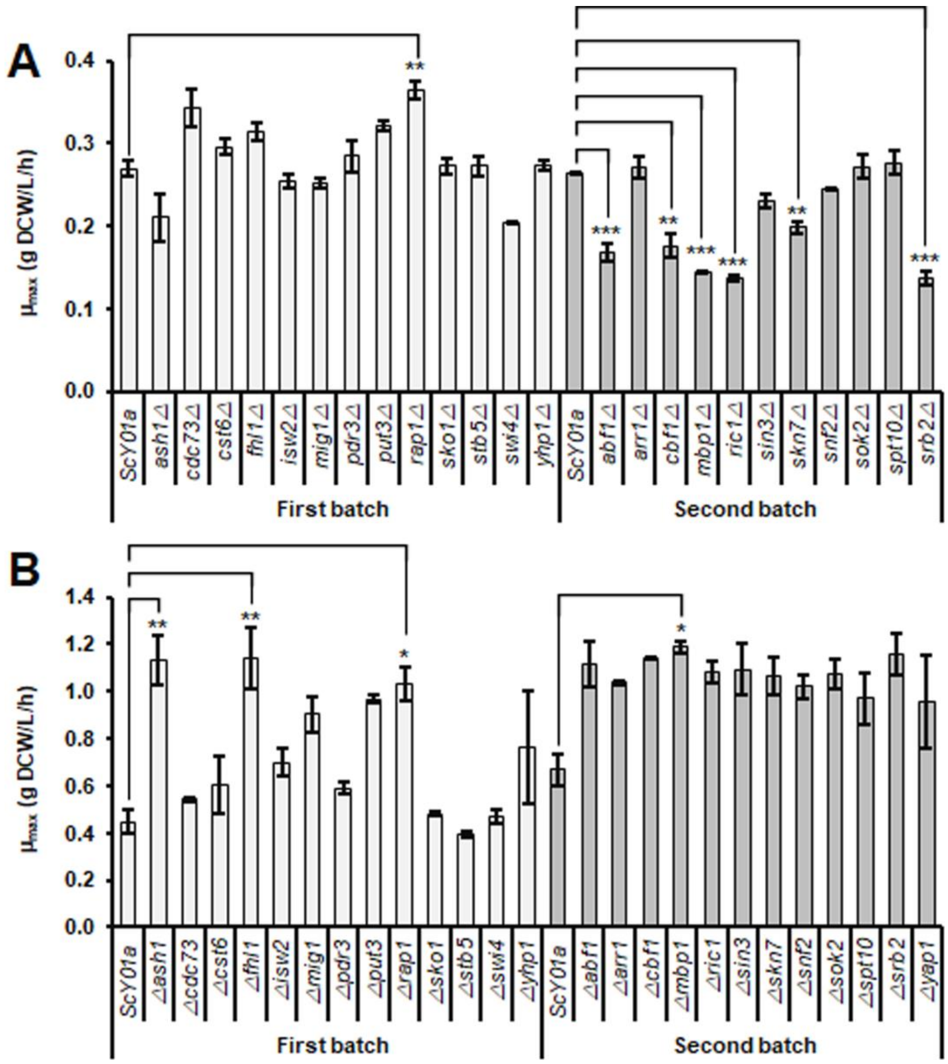

Supplementary Figure 1. Maximum growth rates (μ_{max}) of 23 key TF single deletion strains at elevated (A, 40ºC) and normal (B, 30ºC) temperatures compared with the wild type strain ScY01a. Fermentations were performed in two batches using YP medium containing 200 g/L glucose and initial OD_{600} of 0.5 at 220 rpm. Data represent the mean and standard error of duplicate cultures. Statistical analysis was performed using one-way ANOVA (with strains as the factor) followed by Tukey's multiple-comparison posttest (*P < 0.05, **P < 0.01, ***P < 0.001, compared to the wild type strain ScY01a in each batch).

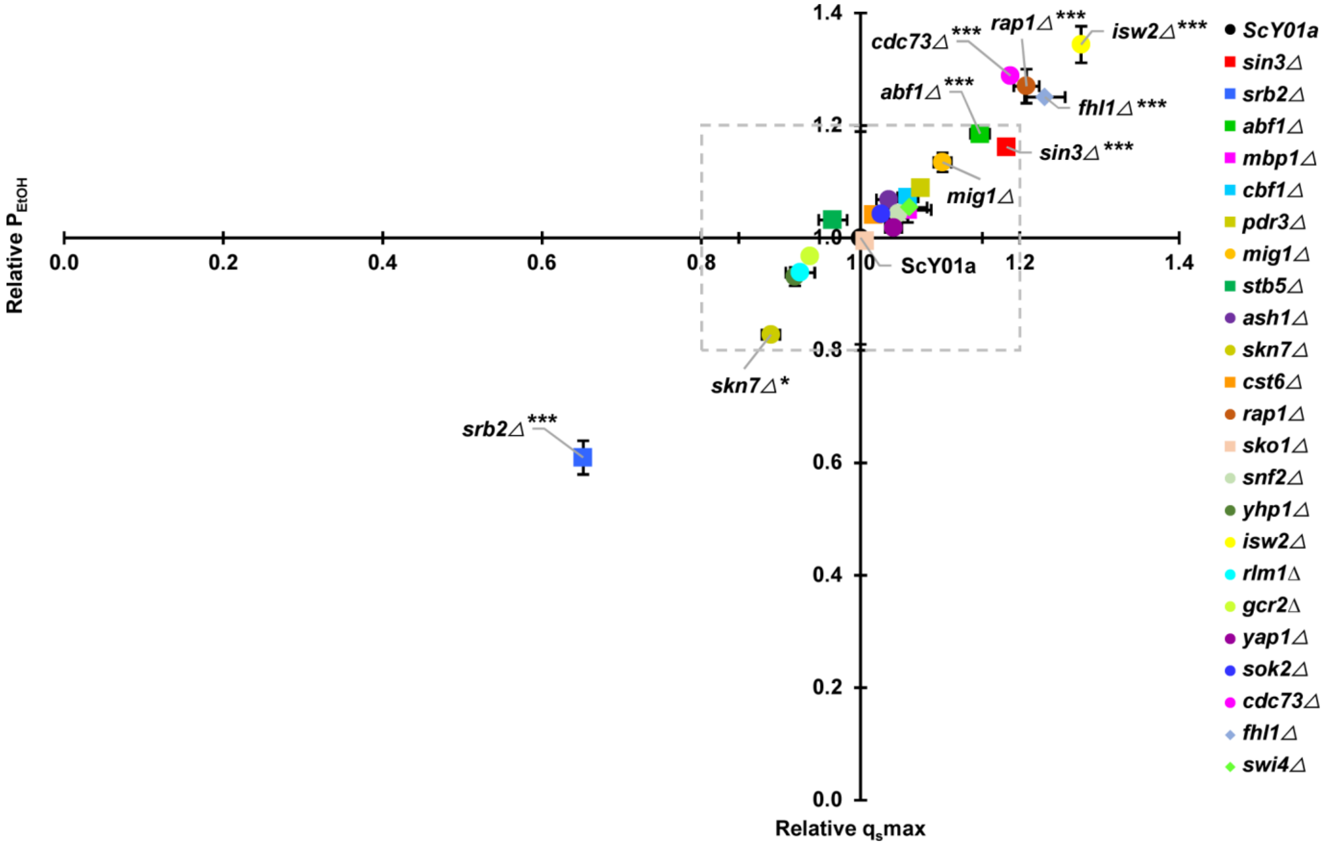

Supplementary Figure 2. Effects of key TF deletions on fermentation rates at normal (30ºC) temperature. The relative glucose consumption rate ($q_s$max) and ethanol productivities (P_{EtOH}) were obtained by comparisons to the wild-type strain ScY01a (normalized to 1.0). Plots of relative $q_s$max versus relative P_{EtOH} are shown. The legends from top to bottom are arranged in descending order of relative $q_s$max at 40ºC. Each point represents duplicate fermentations using a starting glucose concentration of 200 g/L and starting OD_{600} of 0.5. The glucose consumption rate and ethanol productivity for the ScY01a strain at 40ºC were 10.33 (± 0.15) g/L/h and 4.91 (± 0.21) g/L/h, and those at 30ºC were 13.65 (± 2.09) g/L/h and 5.83 (± 1.10) g/L/h, respectively. Statistical analysis was performed using two-way ANOVA (with strains and fermentation rate including $q_s$max and P_{EtOH} as the factors) followed by Tukey's multiple-comparison posttest (*$P < 0.05$, **$P < 0.01$, ***$P < 0.001$).

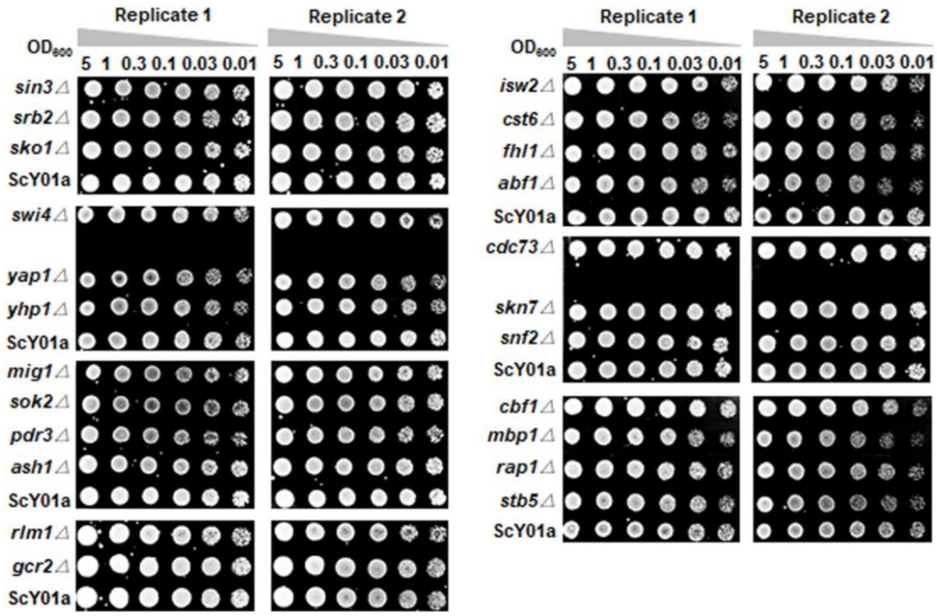

Supplementary Figure 3. Spotted cell growth of key TF deletion strains before heat shock treatment. The 23 TFs deletion mutant strains and the wild type strain ScY01a (Supplementary Table S1) were grown in biological duplicates to early-log phase in YP medium containing 200 g/L glucose (YPD medium). Cells were harvested and resuspended to 5.0 OD_{600} and serially diluted to the OD_{600} indicated and spotted on YPD plates without heat shock treatment. The plates were incubated for 2 days at 30°C and imaged.

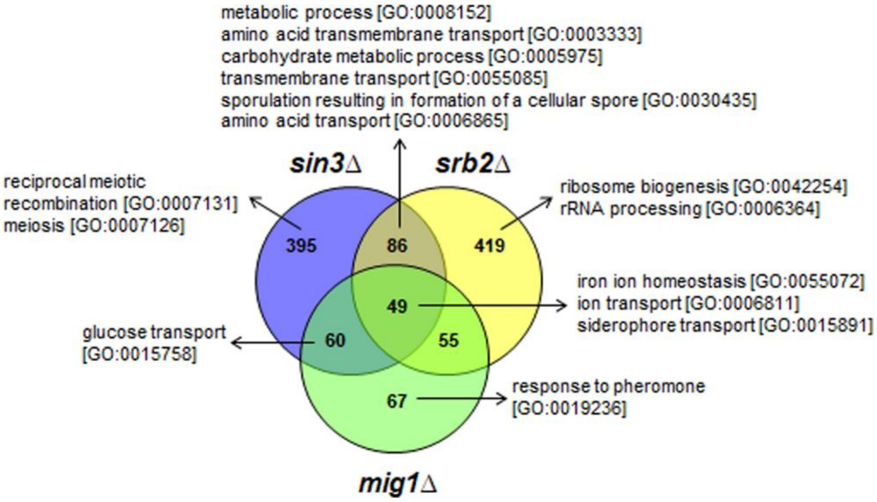

Supplementary Figure 4. Common and specific SDEGs and GO biological processes caused by *SIN3* (in blue), *SRB2* (in yellow) and *MIG1* (in green) deletions.

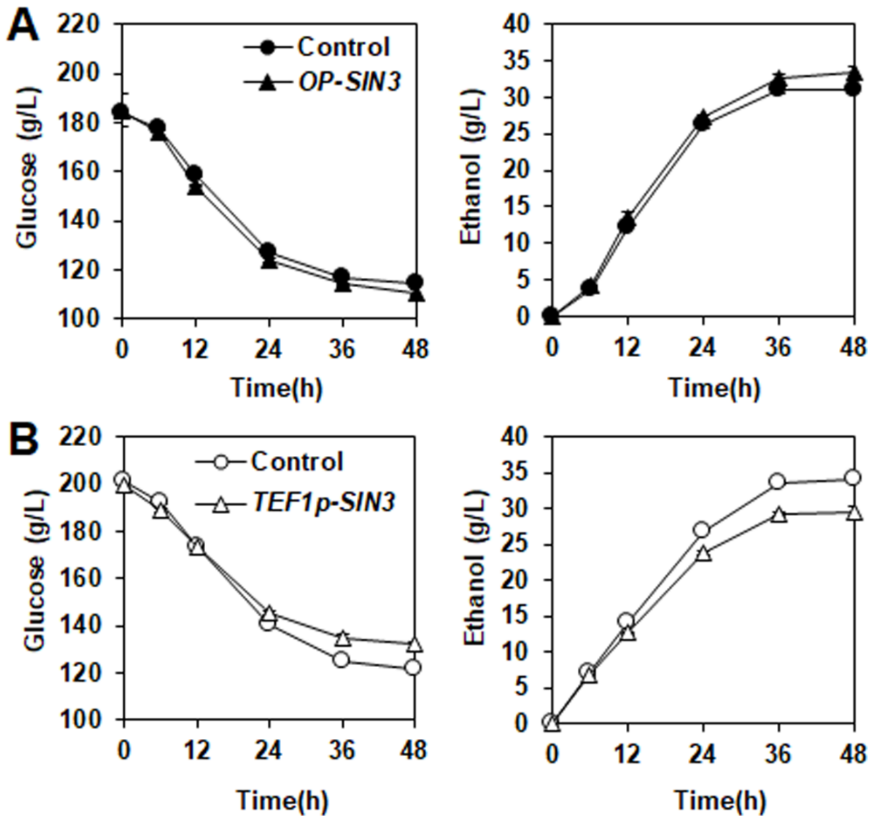

Supplementary Figure 5. Glucose consumption and ethanol production profiles of ScY01a (ura3Δ) and SIN3 overexpression strains using its original promoter (OP) **(A)** and using the strong TEF1 promoter (TEF1p) **(B)**. Fermentation experiment was performed as previously reported (Xiao et al., 2018). Each point represents duplicate fermentations using a starting glucose concentration of 200 g/L and starting OD_{600} = 0.5.
